# Clinical Cysticercosis epidemiology in Spain based on the hospital discharge database: What's new?

**DOI:** 10.1371/journal.pntd.0006316

**Published:** 2018-04-05

**Authors:** Zaida Herrador, Amalia Fernandez-Martinez, Agustín Benito, Rogelio Lopez-Velez

**Affiliations:** 1 National Centre for Tropical Medicine, Health Institute Carlos III (ISCIII in Spanish), Madrid, Spain; 2 Network Biomedical Research on Tropical Diseases (RICET in Spanish), Madrid, Spain; 3 National Referral Unit for Tropical Diseases, Infectious Diseases Department, Ramón y Cajal Hospital, Instituto Ramón y Cajal de Investigación Sanitaria, Madrid, Spain; Negrar Hospital, ITALY

## Abstract

**Background:**

Cysticercosis (CC) is a tissue infection caused by the larval cysts of the pork tapeworm *Taenia solium*. It is usually acquired by eating contaminated food or drinking water. CC Cysts can develop in the muscles, the eyes, the brain, and/or the spinal cord. *T*. *solium* is found worldwide, but its prevalence has decreased in developed countries due to stricter meat inspection and better hygiene and sanitation. Nevertheless, CC is still a leading cause of seizures and epilepsy. In Spain, The disease is not nationally reportable and data on CC infected animals are also missing, despite the European Directive 2003/99/EC.

**Methodology/Principal findings:**

We performed a retrospective descriptive study using the Spanish Hospitalization Minimum Data Set (CMBD). Data with ICD-9 CM cysticercosis code (“123.1”) placed in first or second diagnostic position from 1997 to 2014 were analyzed. Hospitalization rates were calculated and clinical characteristics were described. Spatial distribution of cases and their temporal behavior were also assessed. A total of 1,912 hospital discharges with clinical cysticercosis were identified. From 1998 to 2008, an increasing trend in the number of CC hospitalizations was observed, decreasing afterwards, in parallel with a decrease in the external migration rate. The Murcia region had the highest median hospitalization rate (13.37 hospitalizations/100,000 population), followed by Navarra and Madrid. The 16–44 age group was the most represented (63.6%). The three most frequent associated diagnoses were epilepsy and convulsions (49.5%), hydrocephalus (11.8%) and encephalitis/myelitis/meningitis (11.6%).

**Conclusions/Significance:**

There is a need for a common strategy on data collection, monitoring and reporting, which would facilitate a more accurate picture on the CC epidemiological scenario. Even if most cases might be imported, improving the human and animal CC surveillance will result useful both in gaining extended disease knowledge and reducing morbidity and related-costs.

## Author summary

Cysticercosis (CC), caused by the larval cysts of the pork tapeworm *Taenia solium*, is a neglected tropical disease (NTD). It is acquired when worm eggs are ingested and the developing larvae migrate through the body and form cysts in tissues. Frequent in pigs, it can also affect humans, usually when they swallow *T*. *solium* egg-contaminated soil, water or food (mainly vegetables) or through self-infection or person to person transmission when hygiene practices are insufficient. CC is the most frequent preventable cause of epilepsy worldwide, and is estimated to cause 30% of all epilepsy cases in countries where the parasite is endemic.*T*. *solium* in humans has been emerging as a public health concern in Europe due to the increased number of diagnosed CC cases in recent decades. This situation has been linked to increased travels and migratory movements towards and from endemic countries. In Spain, there is no surveillance system in place. Thus, the CC disease burden remains unknown. This study provides an 18-year review of the epidemiological trends and patient characteristics in order to assess the impact of CC hospitalizations in Spain. In this way, we aimed to describe for the first time the national human CC scenario in Spain.

## Introduction

Cysticercosis (CC) is a parasitic tissue infection caused by larval cysts of the pork tapeworm *Taenia solium* [1]. Pigs, the CC natural intermediate hosts, become infected by eating tapeworm eggs in the feces of a human infected with a tapeworm. Human acquire CC through faecal-oral contamination with *T*. *solium* eggs from human tapeworm carriers [2]. Humans are the only definitive host for *T*. *solium*, while *T*. *saginata* CC is only a disease of cattle and has veterinary importance in beef and dairy production [1,3].

Among foods, uncooked vegetables are the major source [3]. Clinical syndromes related to CC are divided into neurocysticercosis and extraneural cysticercosis [2] [4]. Neurocysticercosis (NCC) is the greatest cause of acquired epilepsy worldwide, and is also increasingly seen in more developed countries because of immigration from endemic areas [2]. Other symptoms of CC include intracranial hypertension, hydrocephalus, meningoencephalitis, psychiatric disorder, stroke, and/or radiculopathy or myelopathy, if the spinal cord is involved [5]. The peak severity of CC has been estimated to occur 3–5 years after initial infection, but it can be delayed over 30 years [6]. Outside the central nervous system, cysticercosis causes no major symptoms besides the eye [2]. Postmortem studies in endemic areas suggest that 80% of NCC infections are asymptomatic. Consequently, many cases are never diagnosed or are found incidentally during imaging procedures [5].

Cysticercosis is a zoonosis of public health importance; with significant economic impacts on the health and meat sectors. The prevalence of CC in humans is highly variable within a country and between countries [1]. This variability is due to hygienic habits and socio-economic conditions, quality of meat inspection and culinary habits. CC is common throughout Latin America, most of Asia, sub-Saharan Africa, and parts of Oceania [7]. In Europe, human CC is not notifiable and therefore it is difficult to assess its epidemiology. Detection and reporting of animal CC cases is mainly based on official meat inspection [8]. CC was considered to be endemic in the past, however, some foci continue to be reported in Bulgaria, Latvia, Lithuania, Poland, Portugal, and Romania [9]. Sporadic cases of CC have also been documented in other EU countries, yet it is not known if transmission occurred recently or in the past. An effective surveillance system is lacking at European level, so the problem may be certainly underestimated [10]. Besides, the increasing number of migrants from endemic countries (specially Latin America, Sub-Saharan Africa and Southeast Asia) and international travelers has resulted in an increase of CC in Europe [11]. A particular epidemiological situation is present in Spain and Portugal, where a recent review carried out by Fabiani et al. showed a number of cases more than fivefold the total cases reported in the seventeen European countries analyzed [10].

Spain is the country reporting the highest number of imported cases of CC, probably because it hosts the largest number of migrants coming from Latin American in Europe [9], where CC is highly prevalent [12]. Up to date, there is no surveillance system for CC disease implemented in Spain. However, hospitalized cases are recorded within the National Health System′s Hospital Discharge Records Database (Conjunto Mínimo Básico de Datos or CMBD in Spanish) belonging to the Spanish Ministry of Health. In this paper, we describe for the first time CC related hospitalizations in Spain between 1997 and 2014, in terms of time, geographical distribution, and disease related individual characteristics.

## Methods

### Data analysis

We performed a retrospective descriptive study using the CMBD for the time period January 1st, 1997 to December 31st, 2014. International Classification of Diseases, Ninth Revision, Clinical Modification (ICD-9CM), the ICD version employed during the study period, was used for this purpose [13]. Registers with ICD-9 CM “other cestode infection” codes (“123.*”) placed in first or second diagnostic position were analyzed. For further analysis, hospitalization discharges coded as cysticercosis (ICD-9 CM code 123.1) were selected.

The CMBD database receives notification from around 98% of the public hospitals in Spain [14]. The National Health System (NHS) provides free medical care to 99.5% of the Spanish population, although those persons not covered by the NHS can be attended at the public hospitals. Private hospitals represent only a small proportion of all hospital admissions. Since 2005, CMBD also has a gradual coverage from private hospitals [15].

For each entry, we collected socio-demographic (sex, age and autonomous community of residency) and clinical data (other diagnosis, type and department of admission, average length of hospitalization, non-invasive procedures and history of surgical intervention during the hospitalization, re-admission, outcome, hospitalization′s cost to the health care system, financing regime and diagnosis related group (DRG)). Age was categorized in four groups: 0–15, 16–44, 45–64 and ≥ 65 years old. These four age categories were selected to provide an overview of children, young adults, older adults and seniors. Other selected co-diagnoses were also explored. These diagnoses were assessed by searching all those ICD-9-CM codes possibly associated with CC in any diagnostic position, and included: diseases of the nervous system and sense organs, endocrine, metabolic and immunity disorders and HIV infection. Differences in proportions were assessed by the χ2 test and 95% confidence intervals (95% CI) were calculated. ANOVA was used to compare differences in means. We used two-sided tests and p < 0.05 was considered significant.

The average number of hospitalizations per year and autonomous community (Comunidades Autónomas or CC.AA in Spanish) were calculated in order to assess temporal and geographical patterns. Official population figures of the Spanish municipalities were used as population at risk for the study period 1998–2014 [16]. Data was missing for 1997, thus the population data from the Intercensus Population Estimates were used for that year [17]. Migrations Statistics were also obtained from the Spanish National Statistical Institute. The external migration rates were computed by using residential variation statistic, only available for the period 2002–2014 [18]. Those countries considered endemic for CC (Latin America, Southeast Asia and Sub-Saharan Africa) were selected. External migration rates were computed for the whole country and separately for those CC.AA with the highest CC hospitalization rates. It was assumed that the age distribution of the population covered by these hospitals was similar to the general population. Results in terms of mean rates by CC.AA were plotted in maps for the whole study period using the Geographical Information System QGis free software version 2.18.13. Data analysis was performed using STATA software version 12.

### Ethics statement

This study involves the use of patient medical data from The Spanish Centralized Hospital Discharge Database (CMBD). These data are hosted by the Ministry of Health Social Services and Equality (Ministerio de Sanidad, Servicios Sociales e Igualdad or MSSSI in Spanish). Researchers working in public and private institutions can request the databases by filling, signing and sending a questionnaire available at the MSSSI website. In this questionnaire a signed Confidentiality Commitment is required. All data are anonymized and de-identified by the MSSSI before it is provided to applicants. According to this Confidentiality Commitment signed with the MSSSI, researchers cannot provide the data to other researchers that must request the data directly to the MSSSI [14].

## Results

### Spatial and temporal trends in Spain

A total of 2,067 hospital discharges with “other cestode infection” diagnosis placed as first or second diagnostic position were identified for the 18-year study period (ICD-9-CM codes 123.*). Out of them, 1,912 cases (92,5%) were cysticercosis hospitalizations, while the remaining 155 hospitalizations were due to intestinal taeniasis (Table 1).

**Table 1:** Hospitalization discharges caused by intestinal and tissue *Taenia spp* (n = 2,067), CMBD database, 1997–2014, Spain.

Type of taeniasis	ICD-9 codes	ICD-9 description	n	%
**Intestinal taeniasis**	123.0	*Taenia solium* infection intestinal form	26	1.26
	123.2	*Taenia saginata* infection	59	2.85
	123.3	Taeniasis unspecified	70	3.39
***Taenia solium* cysticercosis**	123.1	Cysticercosis	1,912	92.50
**Total**			2,067	100

**Fig 1:**
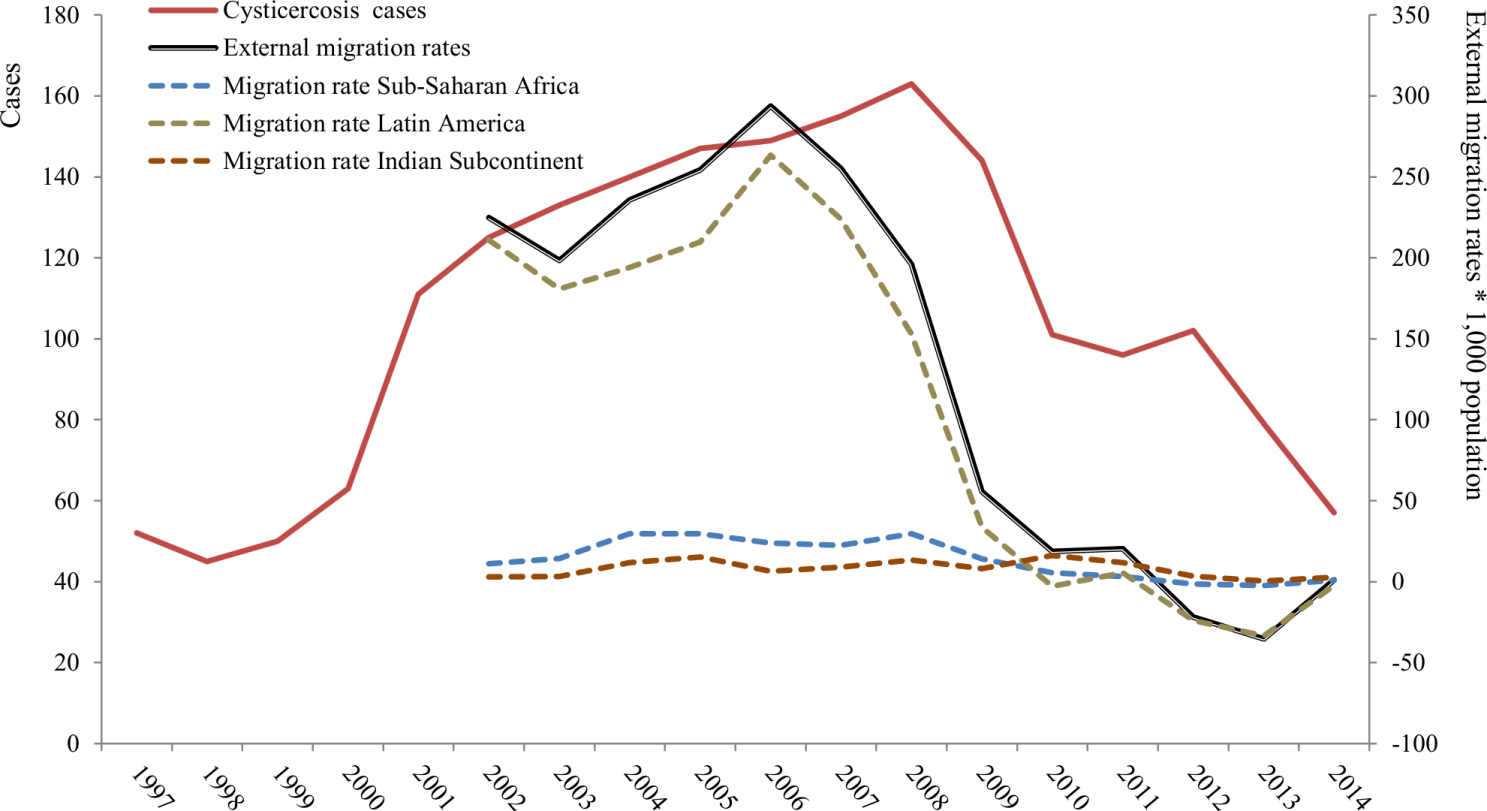
Temporal trend of hospitalizations with cysticercosis as first or second diagnosis and external migration rates.

The temporal distribution of hospitalizations related to clinical CC as first or second diagnosis during the 18-year study period is represented in Fig 1. From 1998 to 2008, an increasing trend in the number of CC hospitalizations was observed. Hospitalization rates peaked in 2008 and were afterwards followed by a steady decline, in parallel with a decrease in the external migration rate, which began to decline in 2006.

**Fig 2:**
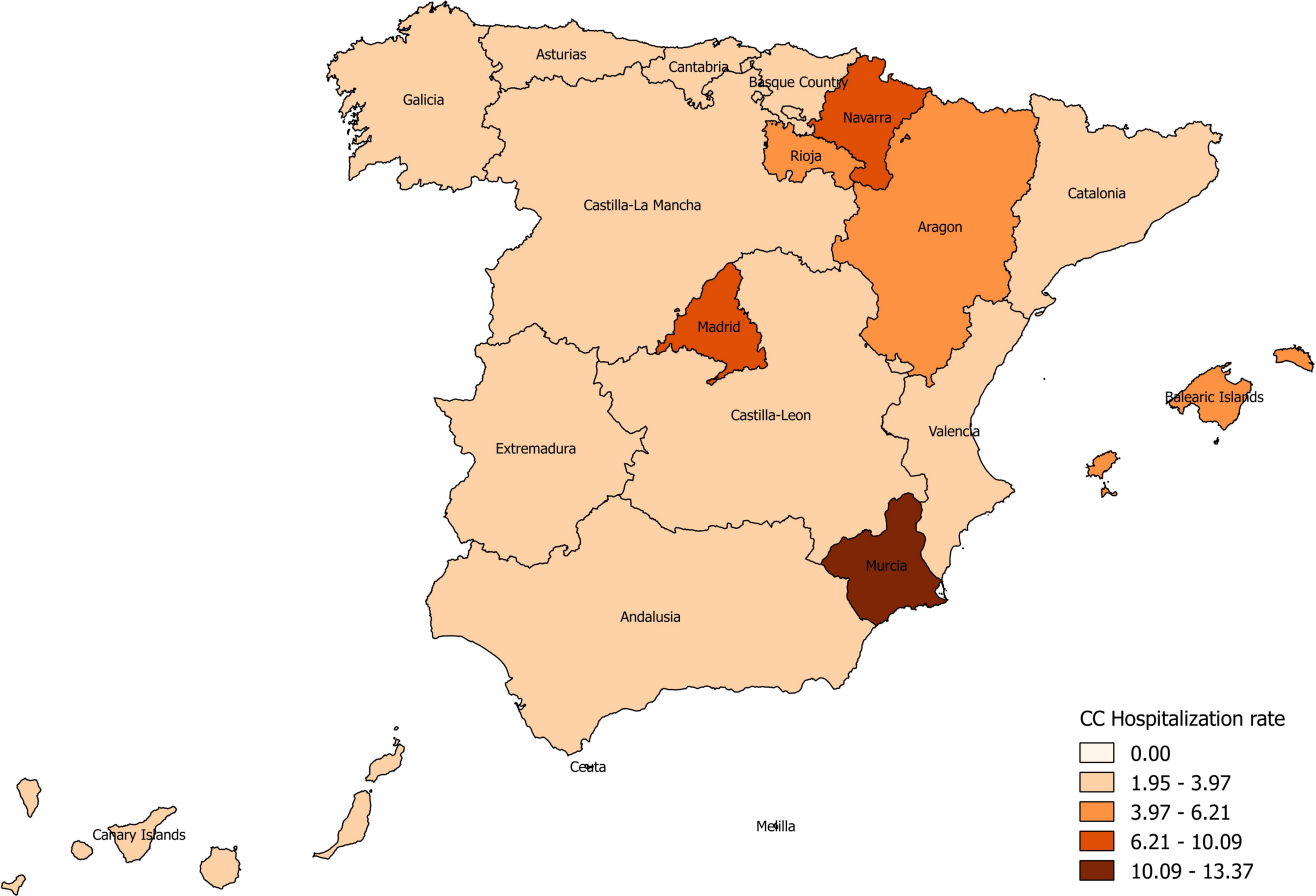
Clinical cysticercosis hospitalizations rates per 100,000 person-years by autonomous community, 1997–2014, Spain.

At national level, the median annual CC hospitalization rate was 4.22/100,000 population. Regarding the regional distribution, the Murcia region had the highest median CC hospitalization rate (13.37 hospitalizations/100,000 population), followed by Navarra (10.09/100,000 population), Madrid (9.32/100,000 population), Aragon (6.21/100,000 population) and Rioja (6.04/100,000 population) (Fig 2, S1 Table).

**Fig 3:**
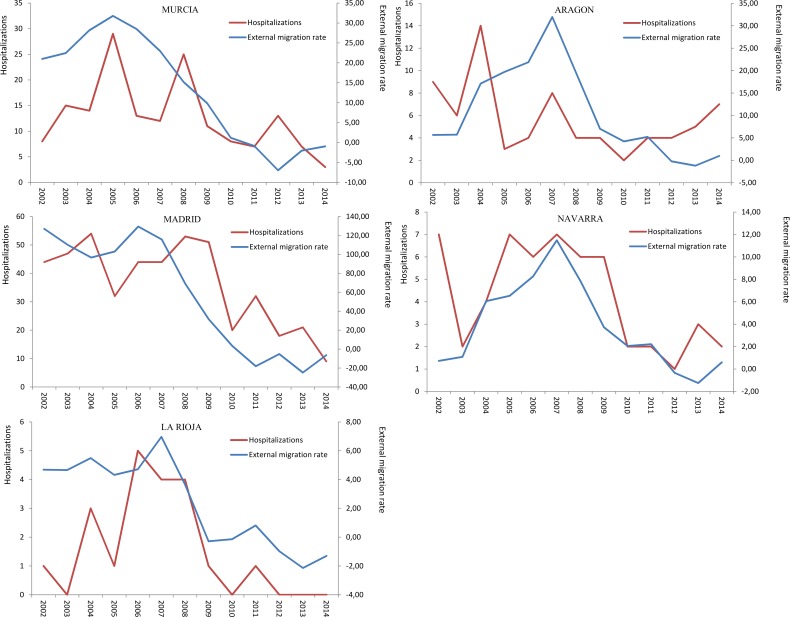
Number of cysticercosis hospitalizations and external migration rates in those regions with higher rates.

Hospitalization trends from those regions with higher rates revealed different patterns. In Murcia, hospitalization rate reached high values in 2005, 2008 and 2012, with an overall decreasing trend. A similar trend was identified in Aragon, with peaks in 2004 and 2007 but a tendency to increase since 2011. In Madrid and Navarra, the tendency in the number of hospitalization rates paralleled the external migration rate. In Rioja there was a peak between 2006 and 2008 (Fig 3).

### Clinical characteristics of cysticercosis related hospitalizations

The mean age of the 1,912 CC hospitalized patients was 38 years (range 0–97), being the 16–44 age group the most represented. This age group also had the highest incidence rate (6.3/100,000), followed by above 65 years old (3.4/100,000), 45–64 years old (3.0/100,000) and under 15 (2.2/100,000). The percentage of CC hospitalized men and women was similar. The majority of patients were discharged at home, decease occurring in 1.5%. The average length of stay for clinical CC hospitalizations was 13 days. We found a wide range for the hospitalization median cost with a median per patient of 29,327.4 euros. Other clinical characteristics are summarized in Table 2.

**Table 2:** Clinical characteristics of cysticercosis hospitalizations (n = 1,912), CMBD database, 1997–2014, Spain.

Variables		n (%)
**Sex**	**Male**	983 (51.4)
	**Female**	929 (48.6)
**Age-groups **	**<15 y**	145 (7.6)
	**16–44 y**	1,216 (63.6)
	**45–64 y**	308 (16.1)
	**> = 65 y**	243 (12.7)
**Surgical intervention**	**No**	1,690 (88.4)
	**Yes**	222 (11.6)
**Type of admission**	**Urgent**	1,558 (81.5)
	**Programmed**	352 (18.4)
	**Others/unknown**	2 (0.1)
**Type of discharge**	**Home**	1,813 (94.8)
	**Transfer**	46 (2.4)
	**Others/unknown**	25 (1.3)
	**Exitus**	28 (1.5)
**Readmission**	**No**	1,707 (89.3)
	**Yes**	205 (10.7)
		**Median (range)**
**Hospitalization time (days)**		13.1 (0–205)
**Hospitalization cost (euro)**		29,327.4 (0–2,455,119.2)

The most frequent diagnostics associated with clinical cysticercosis were diseases of the nervous system and sense organs, such as epilepsy and convulsions (49.5%), hydrocephalus (11.8%) encephalitis/myelitis/meningitis (11.6%) and occlusion of cerebral arteries/hemiplegia (4.2%), which could be related to subarachnoideal NCC.

Of all hospitalizations with CC, surgical procedures in the central nervous system occurred in 520 hospitalizations (27.2%). The most frequent were: diagnostic procedures on spinal cord and spinal canal structures (n = 367), extracranial ventricular shunt and revision, removal, and irrigation of ventricular shunt (n = 169), procedures over the skull (craniotomy), brain, and cerebral meninges (n = 30), and other neurosurgical procedures as ventriculostomy (n = 52).

Regarding other non-neurological diagnoses, the most frequent were unspecified essential hypertension (9.1%), pure hypercholesterolemia (4.5%) and unspecified hyperlipidemia (4.3%), diabetes mellitus (3.8%) and some addictions such as tobacco use disorder (8.7%) and alcohol dependence syndrome (2.3%) (S2 Table).

Major diseases causing immunosuppression were checked in the database. Only 12 patients were seropositive for the HIV and 3 presented disorders of the immune system causing immunosuppression. Comparison of “immunosuppressed” and “immunocompetent” patients´ clinical characteristics was carried out. The exitus outcome was more frequent in the immunosuppressed group (6.7% vs 1.4%), although this difference was not significant (p = 0.092). The only significant difference was related to the hospitalizations time; immunosuppressed patients were hospitalized longer than immunocompetent patients (p<0.001).

## Discussion

This study provides an 18-year review of the epidemiological trends and patient characteristics in order to assess the impact of CC hospitalizations in Spain. Overall, there were few intestinal taeniases compared with clinical cysticercosis. Humans can become infected with taeniasis by eating raw or undercooked beef (*T*. *saginata*) or pork (*T*. *solium* and *T*. *asiatica*) [9]. This lower rate would be explained because *Taenia spp* infections are mostly managed in outpatient care, not requiring hospital admission. On the other hand, T. *solium taeniasis* could be overestimated since *T*. *solium* can be misidentified with the more common *T*. *saginata* [19]. Furthermore, prevalence of taeniosis is usually lower than that of CC, a fact understandable as one tapeworm carrier may infect hundreds of people and thousands of pigs [20].

From 1998 to 2008, an increasing trend in the number of hospitalizations with CC was observed, peaking in 2008 and steady declining afterwards, in parallel with a decrease in the external migration rate. The analysis of the literature showed that due to increased migration and travels from endemic regions, CC is becoming a constantly growing public health problem also in high-income countries, particularly affecting communities where hygiene conditions are poor and consequently the parasite's eggs can spread [4,9,21]. In Spain, and probably related to the economic crisis, many immigrants have return to their country of origin in the last decade, which could contribute to explain the decrease in CC hospitalization rates, at least partially. Unfortunately, the data provided by the Spanish CMBD do not include the place of origin of patients, so this hypothetical relationship is not sufficiently substantiated on data. Autochthonous cases also exist in Europe, particularly in Spain and Portugal [10], but it seems that the patients ‘profile differ. Esquivel et al. reported a growing frequency of CC in Madrid (Spain), especially in young adults with active disease forms, which they related to changes in migratory flows, as Spanish-born patients were elderly, had emigrated many years earlier from rural areas where the disease was formerly prevalent, and had inactive forms of the disease [22]. In our study, the most represented age group was 16–44 years old. This data might stand the hypothesis on the supposed parallel between the number of (hospitalized) cases and the migration trend (in particular that coming from Latin America). Nevertheless, further research is needed to support this hypothesis.

On the other hand, immigration from endemic areas may also play a role in the appearance of autochthonous CC by human to human transmission [2]. CC can be acquired through consumption of food contaminated by a *T*. *solium* tapeworm–infected commercial food handler from endemic countries, or even by direct contact [23]. Taenia eggs, which are infective shortly after been excreted by hosts, have been recovered from the dirt under the fingernails as well as from tapeworm carriers´ skin and clothes [24]. Moreover, tapeworm eggs can survive in contaminated produce for relatively long periods in the environment under favorable conditions [25]. The recent small number of reported cases of autochthonous cysticercosis in the literature may indicate that the risk is low, however, misreporting of the disease and its long incubation period make difficult to assess this transmission pattern.

The 7.6% of clinical CC hospitalizations were pediatric cases (<15 years old). CC can occur in children with only a single exposure to eggs. Children tend to have solitary cysts and fewer complications [26]. In Europe, few pediatric CC have been reported, mainly in migrants from Latin-America [9]. In Spain, a 13 years old autochthonous boy with focal seizure and previous contact with CC infected Ecuadorian has been described [27]. Three more cases in non-Spanish born children have been described in the literature [28,29].

Murcia, Navarra and Madrid were the regions with the highest median hospitalization rates. We know that in Spain, immigrants’ colonies tend to be concentrated within the national territory, mainly in the regions of greater economic weight (Madrid, Catalonia and Valencian community), with relevant differences by country of origin. According to official figures, Madrid and Murcia are among the regions with the highest rates of Latin-American immigration [30]. However, these figures do not explain the case of Navarra. We also found significant differences between regions. When we compare the hospitalization trends within the regions with higher rates, with the external migration rates, only Madrid, Navarra and La Rioja correlated, showing Murcia and Aragón independent patterns. A possible explanation might be the higher proportion of autochthonous cases in these two regions, whether they are truly autochthonous cases, or human to human transmitted. We know that Aragon has recently reinforced the fight against animals’ diseases (2015) [31] and that transmission exist, as confirmed by a recent teenager case in this same region (2017) [32]. Regarding the region of Murcia, it seems that most CC are imported cases [33], even if the graphs didn´t overlapped perfectly. Other possible explanations are differences in disease surveillance over the study period; differences in the interest for cysticercosis from clinicians and researchers, or regional disparities on the diagnostic criteria. Unfortunately, important information is missing in CMBD to settle further hypotheses.

In regard to the signs and symptoms of clinical CC, they are particularly diverse and depend upon several factors, such as the number, location, growth, stage of degeneration and inflammation and host factors [34,35]. In this study, most frequent co-diagnoses were those affecting the nervous system, being the most common seizures/epilepsy/convulsive symptoms, which is consistent with the main associated symptoms to parenchymal CC [3]. Hydrocephalus, encephalitis and meningitis were also common, as well as related medical procedures like ventriculostomy or extracranial ventricular shunt, mainly associated with subarachnoideal and intraventricular CC. Overall, clinical CC symptoms are variable and depend on the types of involvement and degree of inflammation, which in turn may depend on other factors, such as the patient's immune status, exposure time, etc. [35]. Immunosuppression has been linked to the severity of the disease. In fact, the effect of the antiparasitic drug seems to occur by exposing the parasites to the host's immune system, rather than by a direct, immediate drug effect on the parasite as a whole [36]. In this study, few patients presented any disorders of the immune system or were seropositive for HIV. The influence of HIV infection on the natural history of cysticercosis is yet to be defined [37]. Other frequent diagnoses were hyperlipidemia, essential hypertension, tobacco use disorder and diabetes mellitus, although they were less prevalent than in the general Spanish population [38]. Nevertheless, not all the patients´ diagnoses and risk factors are recorded in the CMBD, only those considered by clinicians as related to that hospitalization episode. Moreover, even if all the possible diagnoses were explored in the database, there is important missing information in the CMBD regarding other basal health conditions.

### Limitations and conclusions

Several considerations should be taken into account when interpreting the findings from this research. Our study included cases of CC requiring hospitalization (clinical CC), which is not equivalent to the true CC incidence in the population. The CMBD provides information from a network of hospitals that covers more than 99% of the population living in Spain [14], but it does not include cases managed in outpatient settings or asymptomatic cases, thus CMBD is still underestimating the real burden of CC in Spain. Other relevant limitations in the CMBD are: important individual data such as country of origin or possible related risk factors are missing; information about laboratory test results are not recorded; and the CMBD do not allow the follow-up of patients. Thus, further investigation is recommended.

The use of hospital records data for epidemiological consideration may also be prone to imprecision due to the following reasons. The diagnosis of CC is complicated; imaging findings are rarely pathognomonic and immunodiagnostic tests vary in sensitivity and specificity [39]. Even if the diagnostic criteria have been agreed upon by international experts ‘consensus [40–42], the standard diagnostic criteria in the daily clinical practice may differ.

Finally, at national (and also international) level, data on the animal side of the CC epidemiological scenario are also missing. Despite the European Directive 2003/99/EC which recommends monitoring animal CC according to the epidemiological situation [43], many countries do not report these cases, including Spain [44]. There is a need for a common strategy on data collection, monitoring and reporting, which would facilitate a more accurate picture on the CC epidemiological situation. Even if the majority of cases are imported, improving the human and animal CC surveillance will result useful both in gaining extended disease knowledge and reducing morbidity and related-costs.

## Supporting information

S1 Checklist STROBE checklist. (DOC)

S1 Table Cysticercosis hospitalizations rates per 100,000 person-years by autonomous community, 1997–2014, Spain. (DOCX)

S2 Table Selected co-diagnoses of clinical cysticercosis placed in any diagnostic position (n = 1,656)*, CMBD database, 1997–2014, Spain. (DOCX)
