# Supplementary material for: Clinical Cysticercosis epidemiology in Spain based on the hospital discharge database: What's new?
Source: PLoS Negl Trop Dis. 2018 Apr 5;12(4):e0006316. doi: 10.1371/journal.pntd.0006316 (PMC5886389; doi:10.1371/journal.pntd.0006316)
Supplement: S1 Table — (DOCX) [file pntd.0006316.s002.docx]

| **Supplementary Table 1. Cysticercosis hospitalizations rates per 100,000 person-years by autonomous community, 1997-2014, Spain.** | | | |
| --- | --- | --- | --- |
| **Autonomous community** | **Population average** | **Cysticercosis** | |
|  |  | **Cases** | **Average rate** |
| **Andalusia** | 7,873,481 | 100 | 1.27 |
| **Aragon** | 1,271,451 | 79 | 6.21 |
| **Asturias** | 1,077,071 | 30 | 2.79 |
| **Balearic Islands** | 982,155 | 48 | 4.89 |
| **Basque Country** | 2,138,510 | 72 | 3.37 |
| **Canary Islands** | 1,934,239 | 30 | 1.55 |
| **Cantabria** | 563,194 | 11 | 1.95 |
| **Castilla-La Mancha** | 1,918,806 | 59 | 3.07 |
| **Castilla-Leon** | 2,514,323 | 63 | 2.51 |
| **Catalonia** | 6,949,231 | 276 | 3.97 |
| **Ceuta** | 77,356 | 0 | 0 |
| **Galicia** | 2,758,216 | 97 | 3.52 |
| **Extremadura** | 1,086,704 | 42 | 3.86 |
| **Madrid** | 5,898,538 | 550 | 9.32 |
| **Melilla** | 70,568 | 0 | 0 |
| **Murcia** | 1,323,399 | 177 | 13.37 |
| **Navarra** | 594,473 | 60 | 10.09 |
| **Rioja** | 297,877 | 18 | 6.04 |
| **Valencia** | 4,653,479 | 146 | 3.14 |
| **Total** | **43,983,069** | **1,858*** | **4.22** |
| * 54 Cysticercosis related hospitalizations were recorded as foreigners or unkonwn origin | | | |
