# Supplementary material for: Clinical Cysticercosis epidemiology in Spain based on the hospital discharge database: What's new?
Source: PLoS Negl Trop Dis. 2018 Apr 5;12(4):e0006316. doi: 10.1371/journal.pntd.0006316 (PMC5886389; doi:10.1371/journal.pntd.0006316)
Supplement: S2 Table — (DOCX) [file pntd.0006316.s003.docx]

| **CO-DIAGNOSES** | | **ICD-9 CM codes** | **n** | **%** |
| --- | --- | --- | --- | --- |
| **INFECTIOUS AND PARASITIC DISEASES** | | **001-139** | |  |
|  | Human immunodeficiency virus disease | 42 | 12 | 0.8 |
| **ENDOCRINE, METABOLIC AND IMMUNITY DISORDERS** | | **240-279** | |  |
|  | Pure hypercholesterolemia | 272.0 | 74 | 4.47 |
|  | Other and unspecified hyperlipidemia | 272.4 | 72 | 4.35 |
|  | Diabetes mellitus | 250 | 63 | 3.80 |
|  | Acquired hypothyroidism | 244 | 28 | 1.69 |
|  | Disorders involving the immune system | 279 | 3 | 0.18 |
| **DISEASES OF THE BLOOD AND BLOOD-FORMING ORGANS** | | **280-289** | |  |
|  | Iron deficiency anemias | 280 | 44 | 2.66 |
| **MENTAL DISORDERS** | | **290-319** | |  |
|  | Tobacco use disorder | 305.1 | 144 | 8.70 |
|  | Alcohol dependence syndrome | 303 | 38 | 2.29 |
|  | Tension headache | 307.81 | 21 | 1.27 |
| **DISEASES OF THE NERVOUS SYSTEM AND SENSE ORGANS** | | **320-389** | |  |
| **Inflammatory Diseases Of The Central Nervous System** | | **320-327** | |  |
|  | Encephalitis myelitis and encephalomyelitis | 323 | 133 | 8.03 |
|  | Meningitis due to other nonbacterial organisms classified elsewhere | 321.8 | 30 | 1.81 |
|  | Meningitis of unspecified cause | 322 | 30 | 1.81 |
|  | Intracranial abscess | 324.0 | 16 | 0.97 |
| **Hereditary And Degenerative Diseases Of The Central Nervous System** | | **330-337** | |  |
|  | Obstructive hydrocephalus | 331.4 | 179 | 10.81 |
|  | Communicating hydrocephalus | 331.3 | 17 | 1.03 |
| **Epilepsy and Convulsions** | | **345, 780.3** | |  |
|  | Epilepsy, unspecified, without mention of intractable epilepsy | 345.90 | 301 | 18,18 |
|  | Generalized convulsive epilepsy, without mention of intractable epilepsy | 345.10 | 143 | 8,64 |
|  | Localization-related (focal) (partial) epilepsy and epileptic syndromes with simple partial seizures, without mention of intractable epilepsy | 345.50 | 96 | 5.80 |
|  | Localization-related (focal) (partial) epilepsy and epileptic syndromes with complex partial seizures, without mention of intractable epilepsy | 345.40 | 60 | 3.62 |
|  | Grand mal status | 345.3 | 16 | 0.97 |
|  | Localization-related (focal) (partial) epilepsy and epileptic syndromes with complex partial seizures, with intractable epilepsy | 345.41 | 7 | 0.42 |
|  | Convulsions | 780.3 | 196 | 11.84 |
| **Other Disorders Of The Central Nervous System** | | **340-349** | |  |
|  | Other conditions of brain | 348.8 | 82 | 4.95 |
|  | Cerebral cysts | 348.0 | 60 | 3.62 |
|  | Cerebral edema | 348.5 | 60 | 3.62 |
|  | Migraine, unspecified, without mention of intractable migraine without mention of status migrainosus | 346.90 | 32 | 1.93 |
|  | Benign intracranial hypertension | 348.2 | 26 | 1.57 |
|  | Hemiplegia, unspecified, affecting unspecified side | 342.90 | 21 | 1.27 |
|  | Other conditions of brain | 348.89 | 19 | 1.15 |
| **Mechanical complication of nervous system device, implant, and graft** | | **996.2** | 31 | 1,87 |
| **DISEASES OF THE CIRCULATORY SYSTEM** | | **390-459** | |  |
|  | Unspecified essential hypertension | 401.9 | 150 | 9.06 |
|  | Occlusion of cerebral arteries | 434 | 32 | 1.93 |
|  | Cerebral artery occlusion, unspecified with cerebral infarction | 434.91 | 16 | 0.97 |
|  | Intracerebral hemorrhage | 431 | 14 | 0.85 |
| **DISEASES OF THE RESPIRATORY SYSTEM** | | **460-519** | |  |
|  | Pneumonitis due to inhalation of food or vomitus | 507.0 | 18 | 1.09 |
|  | Pneumonia, organism unspecified | 486 | 16 | 0.97 |
| **SYMPTOMS, SIGNS, AND ILL-DEFINED CONDITIONS** | | **780-799** | |  |
|  | Headache | 784.0 | 57 | 3.44 |
| **SUPPLEMENTARY CLASSIFICATION OF FACTORS INFLUENCING HEALTH STATUS AND CONTACT WITH HEALTH SERVICES** | | **V01-91** | |  |
|  | Presence of cerebrospinal fluid drainage device | V45.2 | 66 | 3.99 |
